# Successful treatment of stellate multiform amelanotic choroidopathy with photodynamic therapy: A case report

**DOI:** 10.1016/j.ajoc.2025.102410

**Published:** 2025-08-19

**Authors:** Luuk van Gorcom, Mehmet Ikinci, Sankha Amarakoon

**Affiliations:** University Eye Clinic Maastricht, MUMC+, P. Debyelaan 25, 6229 HX, Maastricht, the Netherlands

**Keywords:** Stellate multiform amelanotic choroidopathy, Photodynamic therapy, Maculopathy

## Abstract

**Purpose:**

To report a case of stellate multiform amelanotic choroidopathy (SMACH) with focal hyperfluorescence on indocyanine green angiography (ICGA), successfully treated with photodynamic therapy (PDT).

**Observations:**

An 18-year-old male presented with subretinal fluid (SRF) overlying an irregular lesion in the inner choroid. A diagnosis of probable idiopathic macular neovascularization was made; treatment with intravitreal injections (IVIs) of anti-vascular endothelial growth factor (VEGF) agents showed no effect. The diagnosis was later revised to SMACH due to distinctive features on optical coherence tomography (OCT) and ICGA. Typically, no focal leakage is observed on ICGA in SMACH, and no successful treatment has been previously described. However, our case presented with focal leakage on ICGA, and treatment with full-dose PDT led to complete resolution of SRF.

**Conclusions and importance:**

Diagnosing and treating SMACH can be challenging. No effective treatment has been reported to date. We describe a case with focal leakage on ICGA successfully treated with full-dose PDT. We suggest full-dose PDT as the first-line treatment of SMACH with associated focal leakage on ICGA.

## 1 Introduction

Serous chorioretinopathy has a broad differential diagnosis, and establishing the correct diagnosis can be challenging.^1^ Determining the right diagnosis is essential due to possible consequences in treatment and prognosis. Recently, an uncommon macular disorder has been described: ‘serous maculopathy due to aspecific choroidopathy’, later renamed stellate multiform amelanotic choroidopathy (SMACH).^1^, ^2^, ^3^ SMACH is characterized by an irregular choroidal lesion with changes in the retinal pigment epithelium (RPE) on fundoscopy. On optical coherence tomography (OCT) a hyperreflective irregular lesion is seen in the inner choroid, with changes in the overlying RPE. Subretinal fluid (SRF) is present in the majority of cases. Fundus fluorescein angiography (FA) typically shows granular hypofluorescence and hyperfluorescence, late focal staining and leakage is seen in a minority of cases. ICGA shows a delayed filling and late hypofluorescence of the lesion.^3^ Focal choroidal hyperpermeability on ICGA was only recently reported in a single case.^4^ To date less than twenty cases of SMACH have been reported in literature.^2^, ^3^, ^4^ Cases with SMACH can show spontaneous fluctuations in SRF, however no treatment has been shown to affect SRF.^2^^,^^3^ In this paper, we present a case of SMACH with focal leakage on ICGA that has been successfully treated with photodynamic therapy (PDT).

## 2 Case report

**Fig. 1:**
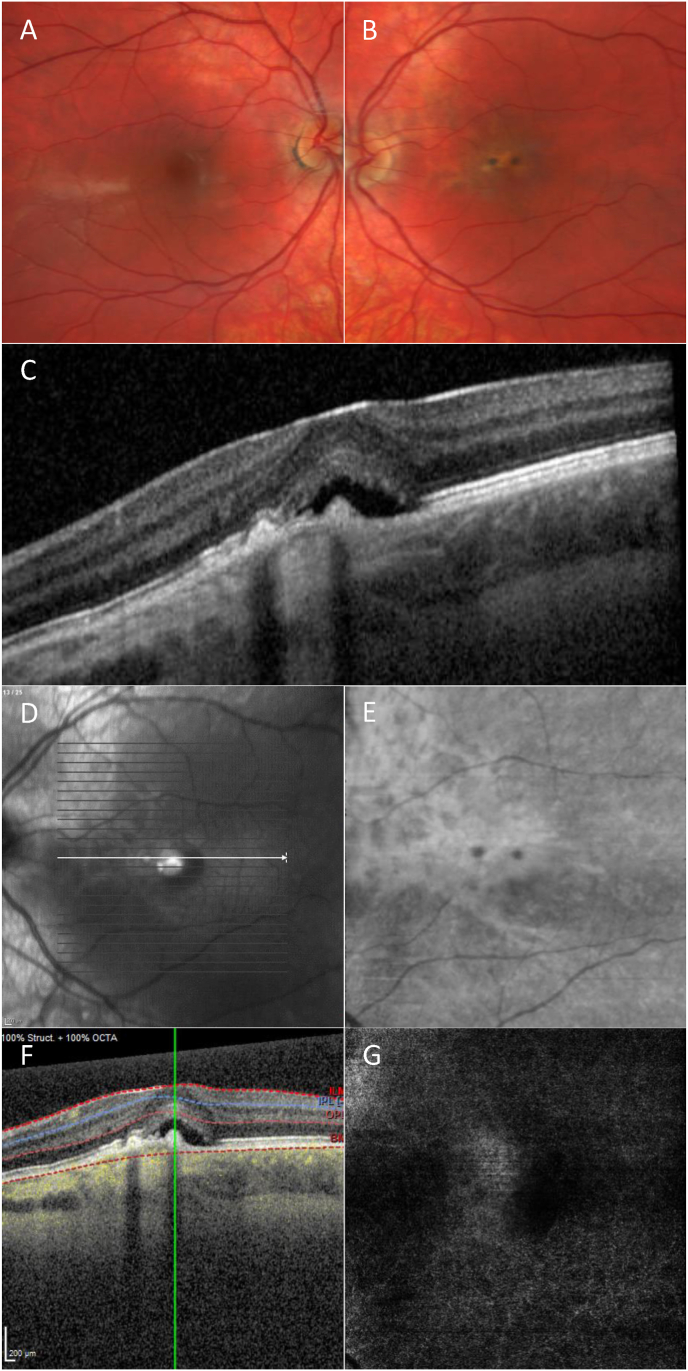
A, Color fundus photos of the right eye shows no abnormalities. B, Color fundus photo of the left eye shows hyper- and hypopigmentation over a choroidal lesion. C, Structural optical coherence tomography (OCT) shows a hyperreflective lesion in the inner choroid, retinal pigment epithelium hypertrophy and subretinal fluid (SRF). D, shows the corresponding scanning laser ophthalmoscopy (SLO) image. E, En face OCT of the choroid shows a hyperreflective stellate lesion with two hyporeflective spots. F,G, OCT angiography reveals no signs of macular neovascularization. (For interpretation of the references to colour in this figure legend, the reader is referred to the Web version of this article.)

**Fig. 2:**
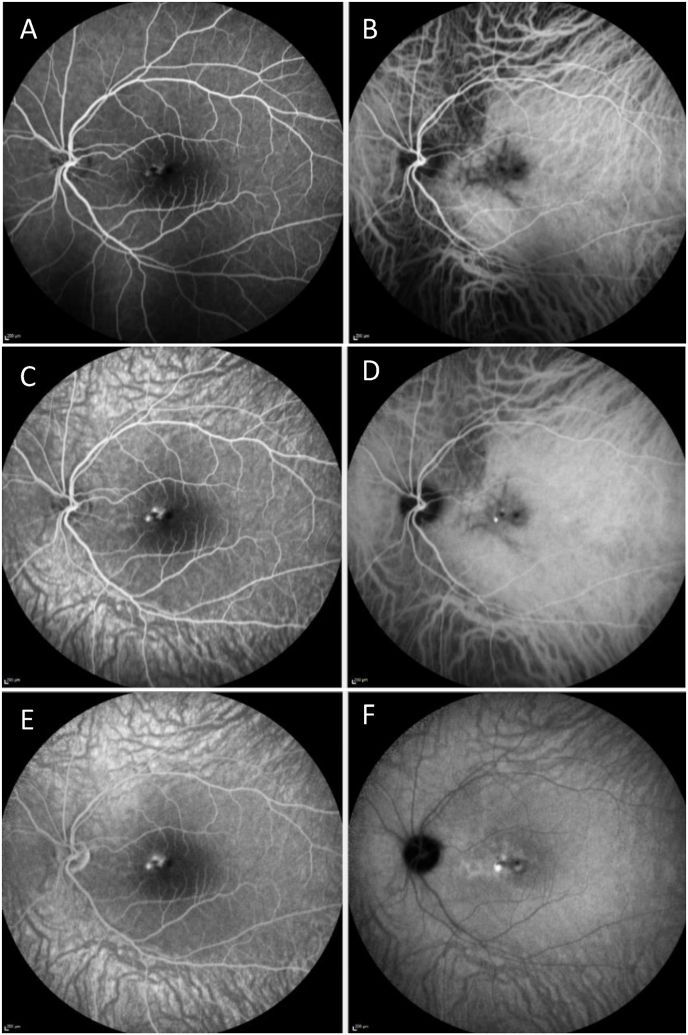
A, Early fundus fluorescein angiography (FA) of the left eye shows hyperfluorescence. B, Early indocyanine green angiography (ICGA) of the left eye shows delayed filling in a stellate pattern. C, Mid-phase FA of the left eye shows multifocal leakage. D, Persistent hypofluorescense in a stellate pattern and focal hyperfluorescence and blockage was seen on mid-phase ICGA of the left eye. E, Late-phase FA of the left eye shows focal leakage. F, Focal leakage was also seen on late-phase ICGA.

An 18-year-old Caucasian male with no relevant medical history was referred to our clinic for evaluation of macular pigmentary changes, SRF and visual acuity (VA) loss in the left eye. Patient had complaints of blurry vision in the left eye since three months, there was no acute onset of symptoms. At presentation, his best-corrected Snellen VA was 1.0 in the right eye and 0.9 in the left eye. His refractive error recorded with autorefractor in diopters (D) was −0.75/-3.75 × 5° in the right eye and −0.50/-4.25 × 171° in the left eye. The astigmatism was regular and no signs of keratoconus were seen on Schleimpflug tomography. Intraocular pressure measured with rebound tonometry was elevated in both eyes, at 25 and 24 mmHg, respectively. Anterior segment examination was unremarkable. Posterior segment examination was normal in the right eye, whereas the left eye showed macular RPE alterations with SRF (Fig. 1A and B). OCT revealed irregular RPE, a hyperreflective lesion in the inner choroid and associated SRF (Fig. 1C and D). En face OCT of the choroid showed a hyperreflective stellate lesion with two hyporeflective spots (Fig. 1E). There was no sign of a macular neovascularization (MNV) on OCT angiography (Fig. 1F and G). FA showed early focal hyperfluorescence with faint leakage in mid- and late-phase. ICGA showed delayed filling and persistent hypofluorescence of the lesion with finger-like protrusions, focal hyperfluorescence was seen in mid- and late-phase (Fig. 2A–C). Since the diagnosis was not clear at that time, we performed additional laboratory test (Appendix A) and ultrasonography, to exclude inflammatory causes, choroidal granuloma, and sclerochoroidal calcification.

**Fig. 3:**
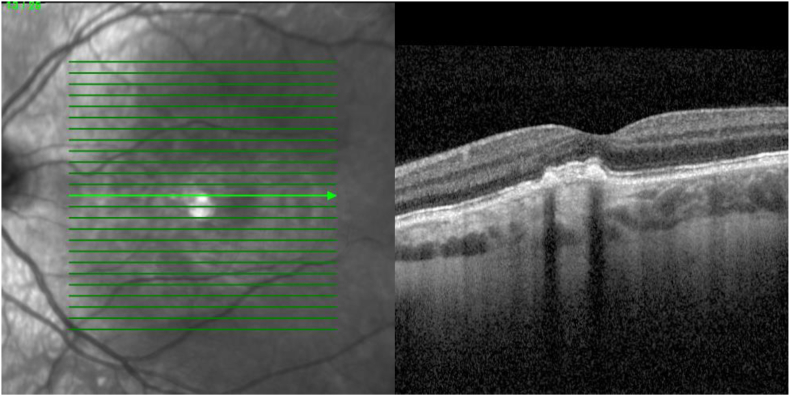
A resolution of subretinal fluid was seen on optical coherence tomography, 10 weeks after photodynamic therapy.

Despite no evident signs of MNV, a single intravitreal injection (IVI) of bevacizumab was administered. Four weeks later, there was no significant decrease in SRF. Subsequently, we administered one IVI with aflibercept 2mg. Upon examination 4 weeks after, reduction of SRF was seen on which 2 additional IVI's with aflibercept 2mg were given with a 4-week interval. No significant decrease of SRF was objectified 4 weeks after the last IVI, Snellen VA declined to 0.7. Shortly after, we were able to revise the diagnosis to SMACH, after a presentation of Ramtohul at the EURETINA Congress 2024.^5^ We opted for PDT since focal leakage was seen on ICGA. Greatest lesion diameter (GLD) was measured 3.7mm on early phase ICGA. Full-dose PDT was chosen since a previous case treated with half-fluence PDT showed no response.^2^ Full-dose PDT (83 seconds, 50j/cm^2^) over the area of leakage with a spot size of 2000μm, based on late phase ICGA, led to a resolution in SRF at 6 and 10 weeks after treatment (Fig. 3). Snellen VA improved to 0.8 and reduction in symptoms were reported.

## 3 Discussion

The pathogenesis of SMACH remains unclear, though it is believed to originate in the choroid.^1^ Considering that SMACH occurs mostly in young patients, it is hypothesized that it is congenital.^3^ Making the diagnosis can be challenging since there is a broad differential diagnosis of serous maculopathies.^1^^,^^3^ Key features differentiating our case from other serous maculopathies are a hyperreflective irregular lesion in the inner choroid on OCT, with delayed filling and late hypofluorescence of the lesion on ICGA with distinctive finger-like protrusions. The spectrum of SMACH may be broader than previously described, as our case is the second reported in the literature to demonstrate hyperfluorescence on ICGA.^3^^,^^4^

Treatment with intravitreal anti-VEGF (aflibercept) showed a reduction of SRF. A possible explanation could be spontaneous fluctuation of SRF over time, which has been described previously.^2^ There were no cases where the SRF in SMACH resolved spontaneously.^2^^,^^3^ Suggesting that PDT contributed to the resolution of SRF in our case.

It is hypothesized that PDT was ineffective in previous cases because no active vascular leakage was observed on ICGA.^2^ In our case there was mid-phase hyperfluorescence on ICGA, which may explain why PDT was effective. Our case might present a subtype of SMACH with focal leakage on ICGA, like the case of Carrera et al.^4^ Moreover, the treatment protocol for PDT was only described in one other case, where half-fluence PDT was used.^2^^,^^3^ This might have influenced the outcomes in these cases.

This case reports on successful treatment with PDT in a patient with SMACH. We suggest full-dose PDT as the first-line treatment of SMACH with associated focal leakage on ICGA.

## CRediT authorship contribution statement

**Luuk van Gorcom:** Writing – review & editing, Visualization, Validation, Supervision. **Mehmet Ikinci:** Writing – review & editing, Writing – original draft, Visualization, Validation. **Sankha Amarakoon:** Writing – review & editing, Supervision.

## Patient consent

Consent has been obtained.

## Authorship

All authors attest that they meet the current ICMJE criteria for Authorship.

## Sources of funding

None.

## Declaration of competing interest

The authors declare that they have no known competing financial interests or personal relationships that could have appeared to influence the work reported in this paper.

## Figures and Tables

**Table:** 

Test	Result	Reference	Unit
Hemoglobin	9.5	8.2–11.0	mmol/L
Hematocrit	0.46	0.41–0.52	L/L
Erythrocyte sedimentation rate	2	0–14	mm
Thrombocytes	239	130–350	10E9/L
White blood cells	5.4	3.5–11.0	10E9/L
Lymphocytes (relative)	26	15–48	%
Monocytes (relative)	7	4–11	%
Eosinophilic granulocytes (relative)	2	0–10	%
Basophilic granulocytes (relative)	0	0–2	%
Neutrophilic granulocytes (relative)	65	40–70	%
Lymphocytes (absolute)	1.4	1.2–5.0	10E9/L
Sodium	142	135–145	mmol/L
Potassium	4.1	3.5–5.0	mmol/L
Total Calcium	2.44	2.10–2.55	mmol/L
Creatinine	85	60–115	μmol/L
eGFR (CKD-EPI)	>90	>90	mL/min/1,73m
Alkaline phosphatase	122	45–120	U/L
Gamma glutamyl transferase	24	<55	U/L
Aspartate transaminase	32	<35	U/L
Alanine transaminase	39	<45	U/L
C-reactive proteine	3	<10	mg/L
Angiotensin-converting enzyme	30	20–70	U/L
Lysozym	9.3	4.8–10.7	mg/L
Soluble IL-2 receptor	533	<600	U/mL
Neopterin	2.3	0.3–2.5	ng/mL
Borrelia burgdorferi IgG and IgM serology	Both negative	–	–
CMV IgG and IgM serology	Both negative	–	–
HSV IgG and IgM serology	Both negative	–	–
VZV IgG and IgM serology	Both negative	–	–
Toxoplasma gondii IgG and IgM serology	Both negatieve	–	–
Treponema pallidum Ig serology	Negative		
TB1 quantiferon	<0.01	–	–
TB2 quantiferon	0.01	–	–

Laboratory test results; eGFR = estimated glomerular filtration rate. CKD-EPI = formula used for eGFR. IgG = immunoglobulin G. IgM = immunoglobulin M. Ig = immunoglobulin. CMV = cytomegalovirus. HSV = herpes simplex virus. VZV = varicella zoster virus. TB = tuberculosis.
